# Complete Genome Sequence of “Candidatus Sulcia muelleri” ML, an Obligate Nutritional Symbiont of Maize Leafhopper (*Dalbulus maidis*)

**DOI:** 10.1128/genomeA.01483-14

**Published:** 2015-01-29

**Authors:** Hsing-Hua Chang, Shu-Ting Cho, Maria C. Canale, Sam T. Mugford, João R. S. Lopes, Saskia A. Hogenhout, Chih-Horng Kuo

**Affiliations:** a Institute of Plant and Microbial Biology, Academia Sinica, Taipei, Taiwan; b Department of Entomology and Acarology, ESALQ/University of São Paulo, Piracicaba, SP, Brazil; c Department of Cell and Developmental Biology, John Innes Centre, Norwich, United Kingdom; d Molecular and Biological Agricultural Sciences Program, Taiwan International Graduate Program, National Chung Hsing University and Academia Sinica, Taipei, Taiwan; e Biotechnology Center, National Chung Hsing University, Taichung, Taiwan

## Abstract

“Candidatus Sulcia muelleri” is a symbiont of sap-feeding insects in the suborder Auchenorrhyncha. The strain “*Ca*. Sulcia muelleri” ML is associated with the maize leafhopper (*Dalbulus maidis*), collected in Brazil, which is a disease vector that affects corn production. Here, we report the complete genome sequence of this bacterium.

## GENOME ANNOUNCEMENT

The bacterium “Candidatus Sulcia muelleri” is a symbiont of sap-feeding insects in the suborder Auchenorrhyncha (1). This bacterium reaches high abundance inside specialized host cells (i.e., bacteriocytes) and provides its hosts with essential amino acids to supplement the nutritional deficiency of plant sap (2–7). The “*Ca.* Sulcia muelleri” genomes characterized to date have a size range of 191 to 277 kb and varied in their gene content for the biosynthesis of seven or eight essential amino acids. For the remaining two or three essential amino acids, their insect hosts have acquired lineage-specific secondary symbionts (4, 6).

To facilitate future comparative and evolutionary analyses, we determined the complete genome sequence of “*Ca.* Sulcia muelleri” strain ML from maize leafhopper (*Dalbulus maidis*). This insect is a vector for three important phytopathogens (i.e., maize bushy stunt phytoplasma, corn stunt spiroplasma, and maize rayado fino virus) that affect corn production in tropical/subtropical America (8). The colony of laboratory-reared insects used in this study was established using specimens collected in Jardinópolis (Sao Paulo State, Brazil; 20.912931 S and 47.896399 W) in 2009. The total DNA of individual insects was extracted using DNeasy blood and tissue kit (Qiagen); 39 samples were pooled for the preparation of Illumina sequencing library.

The procedure for genome sequencing, assembly, and annotation is based on that described in our previous studies (9–15). Briefly, the Illumina MiSeq platform was used to generate 251-bp reads from one paired-end library (~358 bp insert, 3,506,770 reads). The *de novo* assembly was performed using Velvet version 1.2.10 (16) with the following parameters; *k*, 141; scaffolding, no; exp_cov, auto; cov_cutoff, 10; max_coverage, 160; and min_contig_lgth, 2,000. The putative “*Ca.* Sulcia muelleri” ML contigs were identified by BLASTN (17) searches against the “*Ca.* Sulcia muelleri” ALF genome (6). The initial assembly was iteratively improved by mapping the raw reads to the contigs using the Burrows-Wheeler Aligner (BWA) version 0.6.2 (18), programmatically checked using the MPILEUP program in SAMTOOLS package version 0.1.18 (19), and visually inspected using IGV version 2.1.24 (20). All gaps were filled by using reads overhanging at the contig margins. The programs RNAmmer (21), tRNAscan-SE (22), and PRODIGAL (23) were used for gene prediction. For each protein-coding gene, the gene name and product description were initially annotated based on the homologous genes in “*Ca.* Sulcia muelleri” strains ALF (6) and GWSS (2) as identified by OrthoMCL (24). Subsequently, BLASTP (17) searches against the NCBI nonredundant (nr) protein database (25) and the Kyoto Encyclopedia of Genes and Genomes (KEGG) database (26) were used to assist manual curation of the annotation.

The complete genome of “*Ca.* Sulcia muelleri” ML contains one circular chromosome that is 190,405 bp in size with a G+C content of 24.1%. The first version of annotation includes one set of 16S-23S-5S rRNA genes, 29 tRNA genes (covering all 20 amino acids), and 187 protein-coding genes.

### Nucleotide sequence accession number.

The complete genome sequence of “*Ca.* Sulcia muelleri” ML has been deposited at DDBJ/EMBL/GenBank under the accession number CP010105.

## References

[1] Moran NA, Tran P, Gerardo NM 2005 Symbiosis and insect diversification: an ancient symbiont of sap-feeding insects from the bacterial phylum Bacteroidetes. Appl Environ Microbiol 71:8802–8810. doi:10.1128/AEM.71.12.8802-8810.2005. 16332876 PMC1317441

[2] McCutcheon JP, Moran NA 2007 Parallel genomic evolution and metabolic interdependence in an ancient symbiosis. Proc Natl Acad Sci U S A 104:19392–19397. doi:10.1073/pnas.0708855104. 18048332 PMC2148300

[3] McCutcheon JP, McDonald BR, Moran NA 2009 Convergent evolution of metabolic roles in bacterial co-symbionts of insects. Proc Natl Acad Sci U. S. A. 106:15394–15399. doi:10.1073/pnas.0906424106. 19706397 PMC2741262

[4] McCutcheon JP, Moran NA 2010 Functional convergence in reduced genomes of bacterial symbionts spanning 200 My of evolution. Genome Biol Evol 2:708–718. doi:10.1093/gbe/evq055. 20829280 PMC2953269

[5] Woyke T, Tighe D, Mavromatis K, Clum A, Copeland A, Schackwitz W, Lapidus A, Wu D, McCutcheon JP, McDonald BR, Moran NA, Bristow J, Cheng J-F 2010 One bacterial cell, one complete genome. PLoS One 5:e10314. doi:10.1371/journal.pone.0010314. 20428247 PMC2859065

[6] Bennett GM, Moran NA 2013 Small, smaller, smallest: the origins and evolution of ancient dual symbioses in a phloem-feeding insect. Genome Biol Evol 5:1675–1688. doi:10.1093/gbe/evt118. 23918810 PMC3787670

[7] Bennett GM, McCutcheon JP, MacDonald BR, Romanovicz D, Moran NA 2014 Differential genome evolution between companion symbionts in an insect-bacterial symbiosis. MBio 5:e01697-14. doi:10.1128/mBio.01697-14. PMC4196230 25271287

[8] Tsai JH 2008 Corn leafhopper, *Dalbulus maidis* (Delong and Wolcott) (*Hemiptera*: *Cicadellidae*), p 1072–1074. *In* Capinera JL (ed), Encyclopedia of entomology. Springer Verlag, Dordrecht, the Netherlands.

[9] Chung W-C, Chen L-L, Lo W-S, Lin C-P, Kuo C-H 2013 Comparative analysis of the peanut witches’-broom *Phytoplasma* genome reveals horizontal transfer of potential mobile units and effectors. PLoS One 8:e62770. doi:10.1371/journal.pone.0062770. 23626855 PMC3633829

[10] Chung W-C, Chen L-L, Lo W-S, Kuo P-A, Tu J, Kuo C-H 2013 Complete genome sequence of *Serratia marcescens* WW4. Genome Announc 1(2):e00126–13. doi:10.1128/genomeA.00126-13. PMC3622982 23558532

[11] Ku C, Lo W-S, Chen L-L, Kuo C-H 2013 Complete genomes of two dipteran-associated spiroplasmas provided insights into the origin, dynamics, and impacts of viral invasion in *Spiroplasma*. Genome Biol Evol 5:1151–1164. doi:10.1093/gbe/evt084. 23711669 PMC3698928

[12] Lo W-S, Ku C, Chen L-L, Chang T-H, Kuo C-H 2013 Comparison of metabolic capacities and inference of gene content evolution in mosquito-associated *Spiroplasma diminutum* and *S*. *Taiwanense*. Genome Biol Evol 5:1512–1523. doi:10.1093/gbe/evt108. 23873917 PMC3762197

[13] Chang T-H, Lo W-S, Ku C, Chen L-L, Kuo C-H 2014 Molecular evolution of the substrate utilization strategies and putative virulence factors in mosquito-associated *Spiroplasma* species. Genome Biol Evol 6:500–509. doi:10.1093/gbe/evu033. 24534435 PMC3971584

[14] Ku C, Lo W-S, Chen L-L, Kuo C-H 2014 Complete genome sequence of *Spiroplasma apis* B31^T^ (ATCC 33834), a bacterium associated with May disease of honeybees (*Apis mellifera*). Genome Announc 2:e01151-13. doi:10.1128/genomeA.01151-13. 24407648 PMC3886961

[15] Lo W-S, Chen H, Chen C-Y, Kuo C-H 2014 Complete genome sequence of *Vibrio vulnificus* 93U204, a bacterium isolated from diseased tilapia in Taiwan. Genome Announc 2(1):e01005–14. doi:10.1128/genomeA.01005-14. PMC4183885 25278541

[16] Zerbino DR, Birney E 2008 Velvet: algorithms for *de novo* short read assembly using de Bruijn graphs. Genome Res 18:821–829. doi:10.1101/gr.074492.107. 18349386 PMC2336801

[17] Camacho C, Coulouris G, Avagyan V, Ma N, Papadopoulos J, Bealer K, Madden TL 2009 BLAST+: architecture and applications. BMC Bioinformatics 10:421. doi:10.1186/1471-2105-10-421. 20003500 PMC2803857

[18] Li H, Durbin R 2009 Fast and accurate short read alignment with Burrows–Wheeler transform. BioInformatics 25:1754–1760. doi:10.1093/bioinformatics/btp324. 19451168 PMC2705234

[19] Li H, Handsaker B, Wysoker A, Fennell T, Ruan J, Homer N, Marth G, Abecasis G, Durbin R 2009 The Sequence Alignment/Map format and SAMtools. Bioinformatics 25:2078–2079. doi:10.1093/bioinformatics/btp352. 19505943 PMC2723002

[20] Robinson JT, Thorvaldsdóttir H, Winckler W, Guttman M, Lander ES, Getz G, Mesirov JP 2011 Integrative genomics viewer. Nat Biotechnol 29:24–26. doi:10.1038/nbt.1754. 21221095 PMC3346182

[21] Lagesen K, Hallin P, Rødland EA, Staerfeldt H-H, Rognes T, Ussery DW 2007 RNAmmer: consistent and rapid annotation of ribosomal RNA genes. Nucleic Acids Res 35:3100–3108. doi:10.1093/nar/gkm160. 17452365 PMC1888812

[22] Lowe TM, Eddy SR 1997 tRNAscan-SE: a program for improved detection of transfer RNA genes in genomic sequence. Nucleic Acids Res 25:955–964. doi:10.1093/nar/25.5.0955. 9023104 PMC146525

[23] Hyatt D, Chen G-L, LoCascio PF, Land ML, Larimer FW, Hauser LJ 2010 Prodigal: prokaryotic gene recognition and translation initiation site identification. BMC Bioinformatics 11:119. doi:10.1186/1471-2105-11-119. 20211023 PMC2848648

[24] Li L, Stoeckert CJ, Roos DS 2003 OrthoMCL: identification of ortholog groups for eukaryotic genomes. Genome Res 13:2178–2189. doi:10.1101/gr.1224503. 12952885 PMC403725

[25] Benson DA, Karsch-Mizrachi I, Clark K, Lipman DJ, Ostell J, Sayers EW 2012 GenBank. Nucleic Acids Res 40:D48–D53. doi:10.1093/nar/gkr1202. 22144687 PMC3245039

[26] Kanehisa M, Goto S 2000 KEGG: Kyoto Encyclopedia of Genes and Genomes. Nucleic Acids Res 28:27–30. doi:10.1093/nar/28.1.27. 10592173 PMC102409

